# Effects of abdominal emptying and immersion in salt in different concentrations on fatty acids profile and spoilage indices of fish Kotr (*Sphyraena jello*) during freezing

**DOI:** 10.1002/fsn3.1926

**Published:** 2020-10-01

**Authors:** Ali Aberoumand, Farideh Baesi

**Affiliations:** ^1^ Department of Fisheries Behbahan Khatam Alanbia University of Technology Behbahan Iran; ^2^ Msc in Fisheries, Behbahan Khatam Alanbia University of Technology Behbahan Iran

**Keywords:** abdominal emptying, fatty acids profile, immersion in salt, microbial load, *Sphyraena jello*, spoilage indices

## Abstract

Many studies showed that the nutritional value and the fish spoilage changed during handle and processing. In this research, 20 pieces of the Kotr fish were purchased from Behbahan market. After washing the fish, some as whole fish were frozen (sample A) and some were descaled and emptied and without salt frozen (sample B) and some were immersed in saltwater with 4% concentrations (sample C), 8% (sample D) and 12% (sample E) which then frozen. After 30 days of freezing, samples were transferred to the laboratory to measure fatty acids profile, spoilage indices, proximate composition, and microbial load. The results showed that the percentage of saturated fatty acids in whole fish was significantly lower than the other treatments. However, the percentage of omega‐3, omega‐6, and MUFA and PUFA fatty acids in different samples did not show a significant difference, but the ratio between DHA/EPA fatty acids changed significantly. Spoilage indexes in *Sphyraena jello* fillet had a significant difference in process methods. The amount of the indexes decreased with the addition of salt. The addition of salt and the abdominal emptying of *S. jello* resulted in a change in the fat content of the fish fillet, but did not a significant effect on protein, moisture, and ash content of fish fillet. The number of sycrophile bacteria in treatment A was 158 × 10^3^ Cfu/g, which was higher than the other treatments. The lowest level was observed in salted 12% sample. It can be concluded that abdominal emptying and immersion of the Kotr fish in saltwater can lead to preserve the nutritional value and decrease the spoilage indices and increase the shelf life of the product.

## Graphical abstract

Depending on our study, abdominal emptying and immersion in different salt concentrations of *Sphyraena jello* fish during freezing, showed total omega‐3 and PUFA fatty acids in higher values, and TVN and TBA in lower values in salt concentrations 8% and 12%.

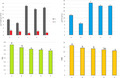

## 1 INTRODUCTION

Fish are considered as an important part in human nutrition, because it containing high contents of polyunsaturated fatty acids (PUFAs), especially of the n‐3 and n‐6 fatty acids series. These unsaturated fatty acids are highly susceptible to oxidation reactions. The packaging samples under vacuum and freezing conditions was a suitable way to reduce the oxidation of the unsaturated fatty acids in the fish Shehri (*Lethrinus microdon*), which cause to extend their shelf life (Aberoumand et al., 2017).

The fish is considered an abundant source of long‐chain PUFAs and valuable nutritional components (Coppes Petricorena, 2015). However, color and lipid oxidation are the main reasons for quality loss in the fish products throughout storage which has near about estimate price of the industry loss annually. The most instances oxidation is lipid oxidation and it is a free radical chain reaction, which can be termed as initiation, propagation, and termination processes (Yogesh et al., 2013). It is a primary mechanism that damages the quality such as the development of off_−_flavor, rancidity, degradation in texture, changing of color in fish and its products during storage, which renders them and made fish unfit for human consumption. These reactions take fish toward loss of nutritive value and eventually declining consumer confidence in the product. Intake of such seafood products that contain lipid components, which are oxidized can modify proteins, tumor initiation, and membrane structure in a biological system (Muik et al., 2005; Porter et al., 1995).

The Kotr fish (*Sphyraena jello*) is one of the most abundant the fish caught in Iran. It is widely consumed which due to its delicious and high consumption and its popularity among local people in Iran. The fresh fish, if well processed and kept at a low temperature, it will reduce the growth of bacteria and other spoilage factors. Primary microbial contamination, storage, and packaging conditions and temperature play an important role in determining the shelf life of fishery products. Khorramgah and Rezaei (2012) studied chemical and sensory changes of whitefish (*Rutilus frisii kutum*) during storage in the freezer at −18°C. Results showed that peroxide value increased until the third month and then decreased. TBA and FFA levels increased during storage. TVB‐N level increased. None of the chemical evaluation indices exceeded at acceptable level. Khoda Nazari and Porashuri (2016) studied chemical, microbial and sensory changes of whole *Otolithes ruber* fish and empty abdominal during storage in ice. His results showed that thiobarbituric acid, free fatty acids, and volatile nitrogen bases levels of whole fish were higher than emptied abdominal fish. Hedayatifard (2003) investigated effects of emptied abdominal of duck fish on its sensory, chemical, and bacterial properties during 3 months of freezing at −18°C. Their results showed that there was no significant difference in the content of moisture, protein, and fat in both groups of whole and emptied abdominal fish. Indices of peroxide, thiobarbituric acid, and total volatile nitrogen in emptied abdominal fish during the storage period increased. The results of a study by Porashouri et al. (2015) showed that TVB‐N level in salted samples of *R. f. kutum* fish was lower than raw samples, which showed inhibitory effects of salt on product spoilage. Shokri et al. (2015) investigated effects of the empty stomach of *rainbow trout* on microbial, chemical, and sensory properties as well as fatty acid profile during storage at −18°C. According to their results, in some cases, moisture content and PV, TBA, and TVB‐N indices were significantly found difference between the two treatments. In the evaluation of fatty acid profile, 15 types of fatty acids were identified, which in both groups and during storage were more than monounsaturated (MUFA) type, followed by saturated fatty acids (SFA) and polyunsaturated fats (PUFA), respectively. During storage, some fatty acid levels showed differences between the two groups. Khoda Nazari and Porashuri (2016) studied chemical, microbial and sensory changes of whole *otolithes ruber* and empty abdominal during storage in ice. Their studies showed that amount of thiobarbituric acid, free fatty acids, and volatile nitrogen bases of whole fish was higher than emptied abdominal fish. The pH of whole salted fish and empty abdominal during ice storage did not show a significant difference. Many studies have been conducted about the effects of salting on the qualitative and chemical properties of fish and other aquatic animals. Hedayati Fard et al., 2011) investigated the effects of salting process on quality characteristics and profile of fatty acids in fish duck during storage in the refrigerator. In this study, the fatty acid profile of fresh and salted fish duck tissue was measured at intervals of 0, 30, 60, and 90 days. Although a large volume of fish blood is excreted by emptying abdominal of fish, which improves color and appearance of fish fillet. The whole fish has a longer shelf life compared to emptied abdominal fish and fillets. Therefore, the whole fish is healthy seafood for a consumer (Joseph & Iyer, 2002). A decrease in volatile nitrogen bases in emptied abdominal fish may be due to reduced bacterial decomposition of nitrogen compounds in fish fillet (Teskeredzic & Pfeifer, 1987). The recommended amount of volatile nitrogen bases in fish fillet is 25 mg nitrogen per 100 g sample as a maximum acceptable level (Nirmal & Benjakul, 2001). According to studies, salt reduces spoilage rate of food products. Using cold saltwater leads to delay in microbiological growth and chemical changes and it creates a good salty taste and makes product more shelf life (Won sik, 2008). The fish salting is one of the traditional methods of fish preservation that is used to reduce spoilage, increase shelf life. Salted products are produced in the world in three ways: products with low salt concentration (1%–8%), medium salt concentration (8%–16%), and products with high salt concentration (Burt, 2004). Although frozen fish can inhibit microbial activity in muscle tissue, its quality changes, which due to various factors such as oxidation of unsaturated fatty acids and causes an unusual odor and taste. These changes, which occur due to hydrolysis and oxidation of fats, are an important factor in the progression of protein denaturation and other tissue changes in fish (Saeed & Howell, 2002). The salting process can be considered as an osmotic treatment that essentially results in sensory and organoleptic properties of the product (Boudhrioua et al., 2009). Studies have been performed on effects emptying the abdominal and intestines on quality changes during the shelf life of fish. Taheri et al. (2012), Studied changes of fatty acids profile of Cobia fish fillet during storage at −18°C. Their results showed that the nutritional value of the fish fillet decreased during storage for 6 months. Khorramgah and Rezaei (2012) examined effects of abdominal emptying on the chemical and sensory properties of white fish during freezing. Results showed changes in quality of emptied abdominal sample found were higher than those of a whole fish during freezing. Similar results were presented by Alaodolei (2013) regard to effects of abdominal emptying of Sof fish in frozen conditions showed qualitative, microbial, and sensory changes during the abdominal emptying process. The effects of abdominal emptying on the chemical, microbiological, and sensory characteristics of farmed bass fish while kept in ice were studied by Papadopoulos et al. (2003). The results of this study showed that shelf life of whole and emptied abdominal sea bass fish was 13 and 8 days based on microbiological and sensory analyzes, respectively. In another study, Lehmann and Aubourg (2007) examined effects of abdominal emptying on the process of rancidity in Cutlass fish during storage at −20°C. The results showed that abdominal emptying of this fish increased rate of oxidation in the frozen product. The researchers suggested that semi oily fish, such as Cutlass fish, do not empty abdominal before freezing.

Brine immersion freezing is used for large fish to be frozen whole, such as salmon and tuna, until they are delivered for sale and processing. This technique developed is extensively used onboard tuna purse‐seine fishing vessels since it allows for efficient fish preservation despite (a) the high seawater temperatures encountered (18–30°C) and the high internal temperature of tunas (Graham et al., 1983; Stevens & Neill, 1978), (b) the high variability in catch rates, and (c) the possibly long‐term storage on tuna fishing vessels (Burns, 1985). The system consists of a saturated sodium chlorine brine immersion tank with a propeller maintained at − 18°C. The brine is circulated around tunas until they are frozen, i.e. between 12 and 48 hr depending on the tank size and catch rate. They are then held for a maximum of 6 weeks either in the refrigerated brine or in dry condition after the brine has been drained from the tank. The rate of salt uptake is driven by technical parameters such as the rise of brine temperature and concentration once fish are immersed and the storage duration (Deng, 1977; Fougére, 1952). These parameters have been extensively investigated by tuna companies to optimize their processes and are strongly controlled aboard vessels to avoid any fish deterioration (Burns, 1985). In addition, some biological factors have been reported to affect salt penetration such as fish species and size, muscle type and composition (Aubourg & Gallardo, 2005; Gallart‐Jornet et al., 2007; Jittinandana et al., 2002).

In the current status, first, the fish was placed in saltwater solution after abdominal emptying and then, analysis of fillet will be done, but in present research, after abdominal emptying of the fish and placing in saltwater solutions with different concentrations, the fish fillet was placed in freezer, and then, the fish fillet was analyzed. In addition, the fish species and salt concentrations and freezing period time in present study were difference comparing the other researches. The necessity of this research is optimization of saltwater concentration and freezing during in preservation of the fish Kotr, so that, its shelf life is longer. Aim of the present study was to evaluate the effects of abdominal emptying and immersion in saltwater different concentrations on fatty acids profile and spoilage indexes of the fish *S. jello* fillet during 30 days of freezing.

## 2 MATERIALS AND METHODS

### 2.1 Preparation of samples

This project was carried out at September 2019 to June 2020 in laboratory of Fisheries Department in Khatam Alanbia University of Technology in Behbahan, Iran. In this research, the twenty the Kotr fish (*S. jello*) were freshly prepared from Behbahan market. These fishes were selected randomly from among healthy fishes. The fishes were placed in box separately in layers of crushed ice with a thickness of approximately five cm and then, were transferred to the laboratory of Fisheries Department, in Khatam Alanbia University of Technology in Behbahan, Iran. First, the fishes were washed; then, some whole fishes were placed in plastic bags and were frozen at −18°C. The remaining samples were emptied. Some of the fishes were immersed in saltwater with different concentrations of 4%, 8%, and 12% and also some fishes were frozen without salt. After 30 days of freezing, the samples transferred to the laboratory in Sari city to analyze of profile of fatty acids, proximate composition such as percentage of protein, fat, ash and moisture, and spoilage indices including peroxide, thiobarbituric acid, free fatty acids, volatile nitrogen and pH values, as well as microbial load (sycrophile bacteria). All materials used to measure the indexes in this project were obtained from Germany Merck Company.

### 2.2 Determination of chemical composition of fish fillet

To analyze the chemical composition, the fish fillet was grinded so that a homogeneous mixture prepared. The sample placed in an oven at 60°C for 24 hr to remove moisture of sample, then dried sample obtained. The samples then powdered by an electric mill and used for chemical analysis in the laboratory in Sari city, Iran.

### 2.3 Measurement of moisture

To determine moisture content, the Chinese dish placed in oven, made in Iran, for 30 min at 103°C to dry. Then, the Chinese dish transferred to a desiccator and weighed after cooling. The 10 g of the sample was put into it and placed in an oven at 103°C for 8 hr. Then, Chinese dish contains sample placed in a desiccator for 30 min and then weighed (measurement was performed for each treatment in triplicate) (AOAC, 2005). The results of each treatment were determined as follows:

Percentageofdriedmaterial=(weightofdriedsample)/(initialweightofsample)×100

Moisturecontent=100‐percentageofdrymatter

### 2.4 Measurement of ash content

The 10 g of the dried sample was placed into the dish, and then, it placed in an electric oven at 500°C for 12 hr. The dish was then brought out from oven and placed in a desiccator for 30 min to cool. The dish was brought out from the desiccator and weighed (AOAC, 2005). The weight of remained material in dish was ash content of the fish fillet, which was determined based on following formula:

Percentageofash=(ashweight/initialsampleweight)×100

### 2.5 Measurement of crude fat content

The 5 g of the sample was weighed by a scale with an accuracy of 0.01 g. The sample was placed in filter paper and, after weighing, was placed in the extractor part of the Soxhlet apparatus, made in Iran. The 100 ml of ether was put into a balloon and the apparatus was connected to refrigerant and the extraction carried out by a heater at 40–60°C for 8 hr. Distillation of solution was continued until balloon was free of solvent (AOAC, 2005). The samples brought out from extractor and were dried in hood.

The fat content of sample was calculated using following equation:

Fat percentage=(initial weight of the sample)-(weight of the sample free fat/initial weight of the sample)×100

### 2.6 Measurement of crude protein content

The protein content of samples determined by Kjeldahl method. To determine protein content in the samples, 10 g of sample was put into a digestion balloon, then, 150 ml of concentrated sulfuric acid was added to each balloon along with the catalyzer. The protein content was determined in triplicate. After placing the balloons in the apparatus, the sample first boiled at low temperature for about 30 min to remove foam and then, temperature increased to digest sample. Digestion of sample took time about 4 hr. After digestion of samples and cooling them, the distilled water added to each balloon and placed in titration section of the Kjeldahl apparatus and titrated with 0.1 N sulfuric acid. Total nitrogen was determined by the Kjeldahl method and then multiplied by 6.25 (AOAC, 2005). The obtained number showed as crude protein content.

Percentageofnitrogen=amountofacidconsumption0.1normal/sampleweight×100

Crudeprotein=percentageofnitrogen×6.25

### 2.7 Measurement of fatty acids profile

#### 2.7.1 Extraction of fillet fat

Folch et al. (1957) used the method to extract fat. The 1 g of the sample transferred to a 50 ml volumetric flask, then 5 ml chloroform added to the sample and shaken vigorously. Then 10 ml of chloroform was added to the sample and the container was shaken vigorously again. The sample was placed at 4°C for 12–24 hr completely to remove the fat. After 24 hr, the samples were transferred into the decanter and 5 ml distilled water was added to it and transferred to the decanter. After 1 hr, separately three phases was formed inside the decanter, the fat and solvent phase, was located in the lower part of the decanter, and was transferred to the COD containers by funnel and filter paper, and by liquid nitrogen, the solvent was separated and the fat remained and measured.

#### 2.7.2 Esterification of extracted fat

The 5 ml of 0.02% methanolic NaOH (2 g of NAOH per 100 ml of methanol) was added to tube contains extracted fat. Then, 1 ml of standard internal solution (a concentration of 2 mg/ml) added, then, the tube closed and shaken vigorously and placed in a boiling water bath for 10 min to perform methylation reflex. Then, it cooled in ambient temperature, and 1 ml of normal n‐hexane added and was shaken. Then, 1 ml of saturated salt (30 g of Nacl in 100 ml of distilled water) was added. The obtained solution was shaken vigorously and settled. After formation two separate phases, upper phase was carefully separated and transferred to 1.5 ml Falcons. 0.2 μl from obtained extract was injected into GC. By comparing inhibition time of chromates of unknown samples with chromatograms obtained from standard solution of methyl ester fatty acids, fatty acids in fish tissue were identified and results were reported as a percentage. To identify fatty acids of samples, it was used as gas chromatograph (GC, made in Germany) CP‐4600 Varian and equipped with BPX70 capillary column (ID: 0.25 mm × 0.22 mm µ 30 m) and flame ionization detector (FID). The detector temperature and injection site were set at 300°C and 250°C, respectively.

### 2.8 Determination of spoilage indexes

#### 2.8.1 Determination of free fatty acids composition

To measure free fatty acids, 25 cc of neutralized ethyl alcohol with normal NaOH was added to oil sample (due to evaporation of remain solvent in down phase of decanter). Amount of free fatty acids was determined as amount of consumed normal NaOH during titration in presence of phenolphthalein and to percentage of oleic acid (Egan et al., 1997).

#### 2.8.2 Determination of thiobarbituric acid content

The TBA content in fish muscle was measured according to Pearson (1976) method. To determine thiobarbituric acid content in fish muscle, 10 g minced sample was weighed and transferred to a distillation balloon (digestion) and 50 cc distilled water was added and stirred for 2 min. 47.5 cc distilled water with 2.5 cc 4 N hydrochloric acid was added again. Digestion was continued until 50 cc distilled solution was obtained. Then, 5 cc the distilled solution was transferred to test tube and 5 cc thiobarbituric reagent (obtained by dissolving 288.3 mg thiobarbituric in 100 cc 90% glacial acetic acid) was added. Test tubes were placed in a water bath at 100°C for 35 min, and then, cooled in cold water for 10 min, and finally absorbance was read at 538 nm using a spectrophotometer, made in England. Each absorbance was used to calculate the TBA value (Dandago et al., 2004).

#### 2.8.3 Determination of pH

10 g of fish sample was completely crushed and homogenized in 10 ml of distilled water. Then, using a digital pH meter, pH of samples was measured (Mahmoud zadeh et al., 2010).

#### 2.8.4 Determination of peroxide number

To measure peroxide number of samples, fish fillets along dried ice were sent to Sari University laboratory after freezing. 50 g of sample placed into 500 ml Erlenmeyer flask and add 200 ml of chloroform to container. The Erlen was shaken for 2 hr to extract. Then, contents of Erlenmeyer were filtered and solution under filter was transferred to abrasive door Erlenmeyer flask. To evaporate solvent, samples were transferred to a rotary evaporator, and after evaporation of solvent, weight of remained oil in Erlenmeyer was determined. For measuring the peroxide content according to AOAC method (2005), extracted oil was dissolved in 30 ml of chloroform and acetic acid mixture and 0.5 ml of saturated potassium iodide added to mixture and mixture was shaken vigorously for one min ( AOAC, 2005). Then, 30 ml of distilled water was added to mixture. After complete mixing, mixture was titrated with 0.01 N sodium thiosulfate solution until light yellow appeared. Then, 0.5 starch reagent of 0.01 N was added to mixture and color of mixture turned dark blue. The titration continued until the blue color was removed and a light color appeared.

#### 2.8.5 Determination of nitrogen volatile material content

The minced fish sample was placed in a balloon contain 2 g of magnesium oxide and 300 ml of distilled water and boiling stone. The distilled vapors were transferred to a 2% solution of boric acid contain a few drops of methyl red reagent and bromocresol green and finally titrated with 0.1 N sulfuric acid. The amount of volatile nitrogen was calculated according to below relationship (AOAC, 2005):

Amountofvolatilenitrogen=ConsumedN0.1sulfuricacid×14

### 2.9 Counting of sycrophile bacteria

To count sycrophile bacteria, agar tryptic medium (TSA) was used by placing 0.1 ml of sample on culture medium and counting bacteria after 10 days of incubation at 4°C (McFaddin, 2000).

#### 2.9.1 Statistical analysis method

In order to statistically analyze the data, first using SPSS software and Kolmogorov‐Smirnov test to determine the normality of data distribution and then using the test (One‐way ANOVA) the presence or absence of differences between the samples was found. After observation of significant difference, Duncan's 95% confidence level was used to test the significance of the difference between the samples.

## 3 RESULTS AND DISCUSSION

### 3.1 Total saturated fatty acids (SFA)

**Figure 1:**
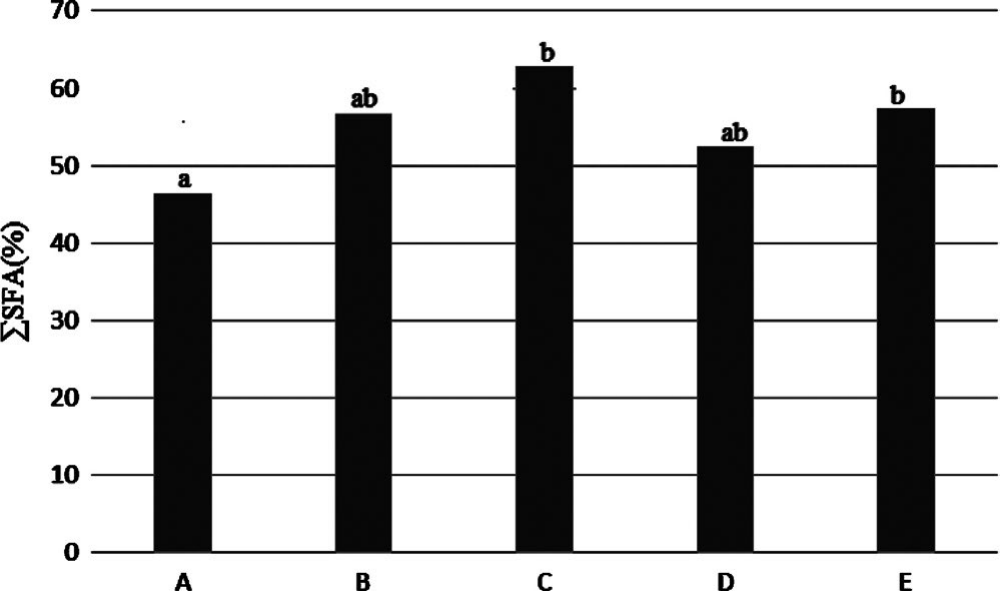
Percentage of saturated fatty acids in the Kotr fish fillet in all treatments during freezing

The results obtained in Figure 1 showed that immersion of the fish in saltwater leads to an increase in total saturated fatty acids in fish tissue. The percentage of saturated fatty acids during freezing in sample C (4% salt) had highest percentage. The lowest was found in whole fish (A).

### 3.2 Total unsaturated fatty acids with a double bond (MUFA)

**Figure 2:**
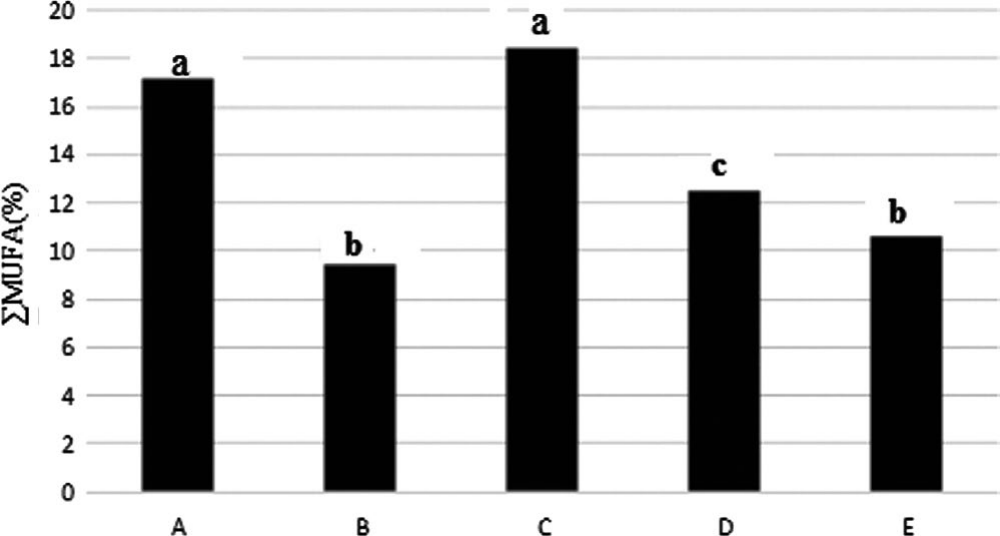
Percentage of unsaturated fatty acids with a double bond in the Kotr fish fillet in all treatments during freezing

The results in Figure 2 stated that percentage of MUFA fatty acids in free salt washed fish (B) had lowest comparing to the other treatments. Highest percentage was found in sample C (salt 4%).

### 3.3 Total polyunsaturated fatty acids (TPUFA)

**Figure 3:**
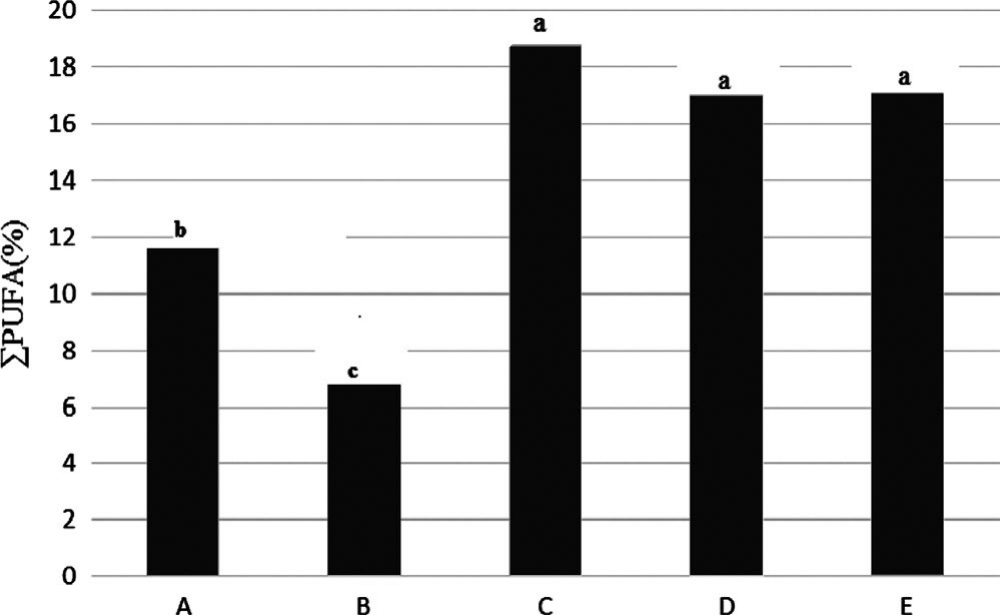
Percentage of unsaturated fatty acids with several double bonds in the Kotr fish fillet in treatments during freezing

The results in Figure 3 showed that addition of salt to the Kotr fish during freezing, increased percentage of unsaturated fatty acids with several double bonds in the fish tissue. Lowest percentage of PUFA fatty acids was found in free salt washed sample (B), but treatment C had highest.

### 3.4 Omega 3 and omega 6 fatty acids

**Figure 4:**
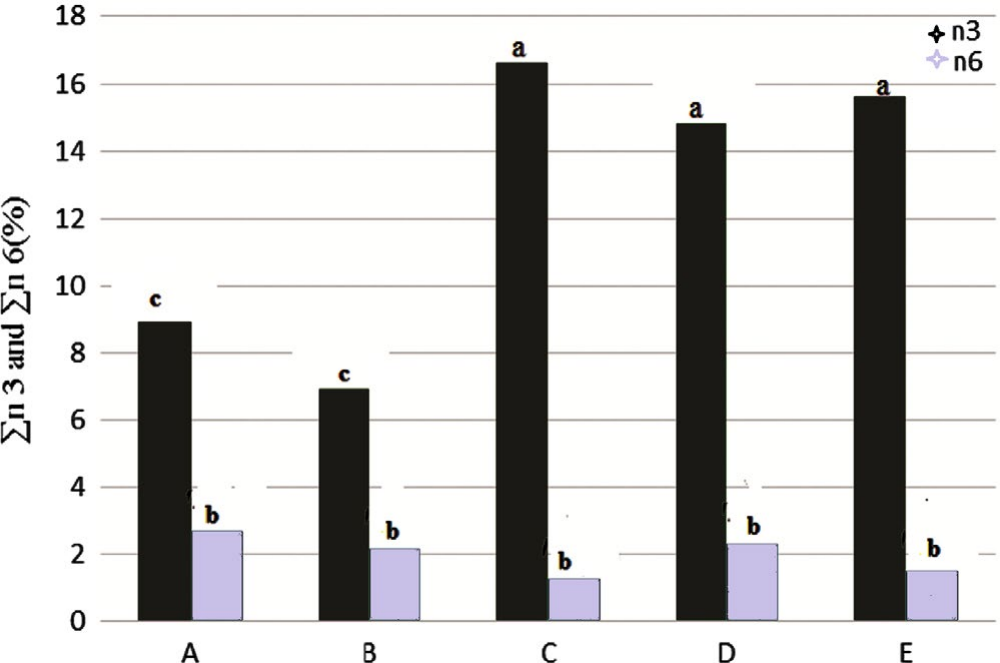
Total omega‐3 and omega‐6 fatty acids in the Kotr fish fillet in all treatments during freezing

Percentage of omega‐3 fatty acids in the fish immersed in saltwater with different concentrations comparing to the other treatments showed a significant increase (*p* < .05) (Figure 4). Lowest percentage belonged to sample B (without salt washed fish) and the sample C (salt 4%) had highest percentage. Percentage of omega‐6 fatty acids in the samples immersed in saltwater with different concentrations was lower than in whole fish. Highest levels of omega‐6 fatty acids were observed in sample A (whole fish), sample D (8% salted) and sample B (without salt washed fish), respectively. Lowest percentage belonged to sample C (salt 4%).

### 3.5 n3/n6 ratio and DHA/EPA ratio

**Figure 5:**
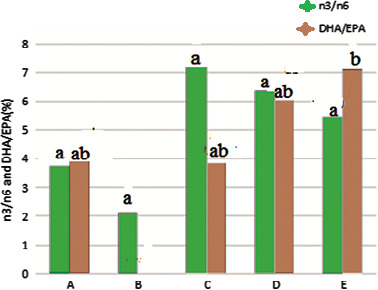
The n3/n6 and DHA/EPA ratios in the Kotr fish fillet in all treatments during freezing

Figure 5 showed that n3/n6 fatty acids ratio in the samples immersed in saltwater with different concentrations was higher than the other treatments. The Kore fish with salt 4% had highest rate. DHA/EPA ratio was high in samples containing the salt, comparing to the other samples (*p* < .05).

### 3.6 PUFA/SFA ratio

**Figure 6:**
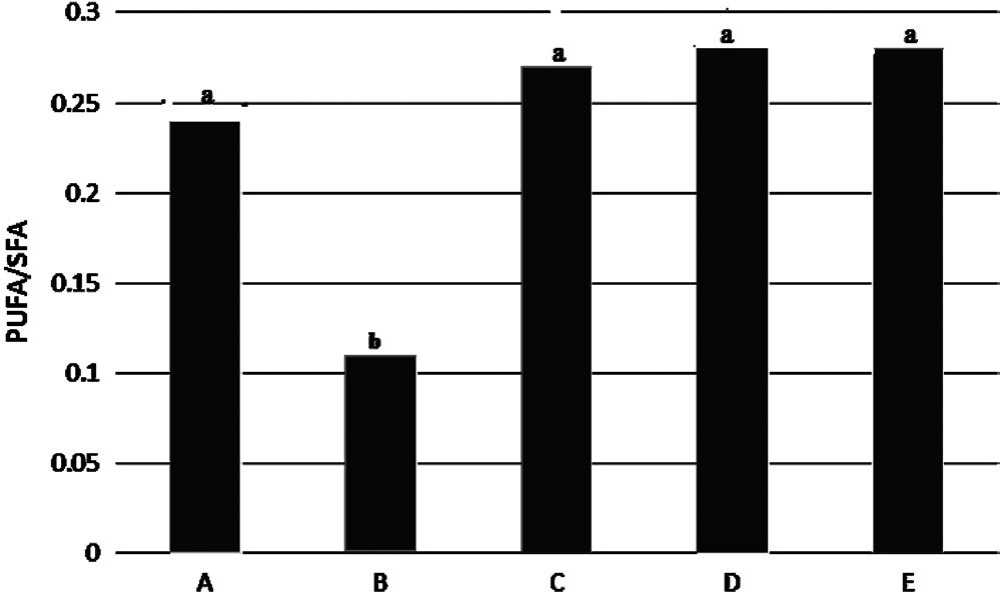
PUFA/SFA ratio of the Kotr fish fillet in all treatments during freezing

Figure 6 showed that there was no significant difference between PUFA/SFA ratio in all treatments. Lowest ratio was reported in sample B and samples D and E had highest.

### 3.7 Spoilage indexes

Results showed the spoilage indices in the Kotr fish during freezing in Table 1. The different methods led to a change in rate of spoilage indexes in the Kotr fish, which in some cases had significant difference with the other samples (*p* < .05).

**Table 1:** The spoilage indexes in the Kotr fish fillet during freezing at −18°C

Spoilage indexes	pH	TBA (mgMDA/kg)	TVN (mgN/100 g)	FFA (%)	PV (meq/kg)
A treatment	5.86 ± 0.18^ab^	0.74 ± 0.02^a^	12.81 ± 0.40^a^	0.46 ± 0.04^a^	0.90 ± 0.02^a^
B treatment	6.08 ± 0.85^a^	0.67 ± 0.00^b^	11.25 ± 0.18^b^	0.45 ± 0.01^ab^	0.85 ± 0.00^b^
C treatment	5.70 ± 0.70^b^	0.63 ± 0.00^c^	11.45 ± 0.20^b^	0.41 ± 0.05^b^	0.91 ± 0.02^a^
D treatment	5.76 ± 0.03^ab^	0.61 ± 0.00^d^	11.00 ± 0.33^bc^	0.39 ± 0.01^b^	0.84 ± 0.02^b^
E treatment	5.62 ± 0.37^b^	0.59 ± 0.01^a^	10.61 ± 0.38^c^	0.35 ± 0.01^c^	0.77 ± 0.02^c^

Note

Sample A, was frozen whole fish after washing, sample B, was for emptied wastes fish and frozen without saltwater, and samples C, D, and E for emptied wastes fish in saltwater with 4%, 8% and 12% concentration, which frozen respectively. Similar letters in each row indicated no significant difference (*p* < .05).

**Figure 7:**
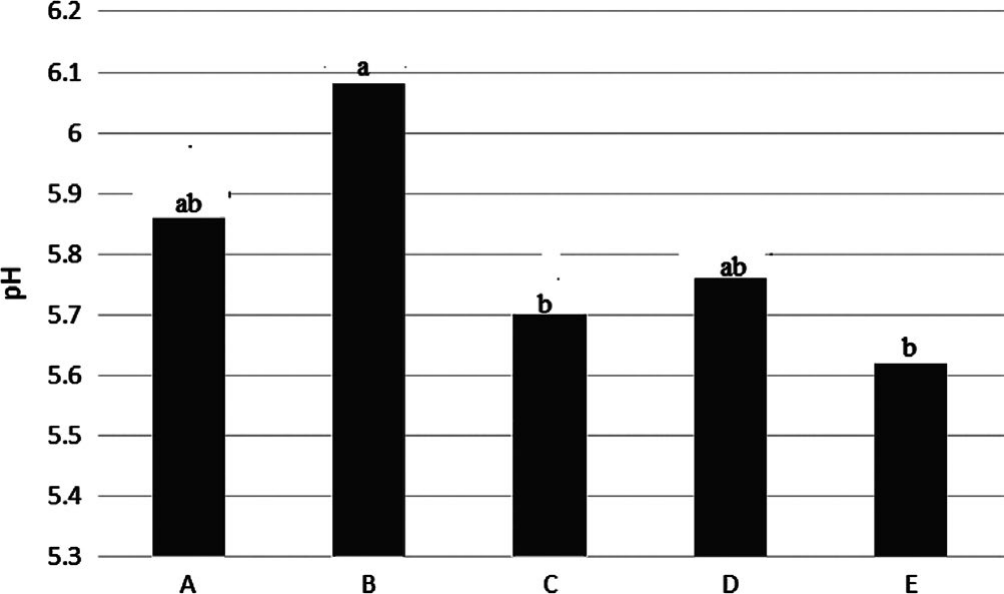
PH value in The Kotr fish in all treatments during freezing

Table 1 and Figure 7 showed that, pH in the Kotr fish sample B during 30 days frozen was highest and sample E had lowest. Samples C and E had a significant difference with sample B and there was no significant difference with the other samples (*p* < .05).

**Figure 8:**
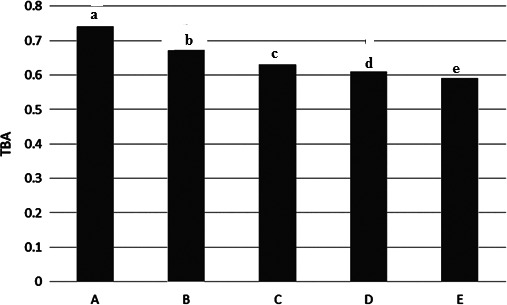
TBA content in the Kotr fish in all treatments during freezing

Table 1 and Figure 8 showed that, level of thiobarbituric acid index in all samples with each other had significantly different (*p* < .05). Highest value showed in whole fish (A) with 0.74 ± 0.02 mgMDA/Kg and lowest value found in 12% salted fish (E) with 0.59 ± 0.01 mgMDA/Kg.

**Figure 9:**
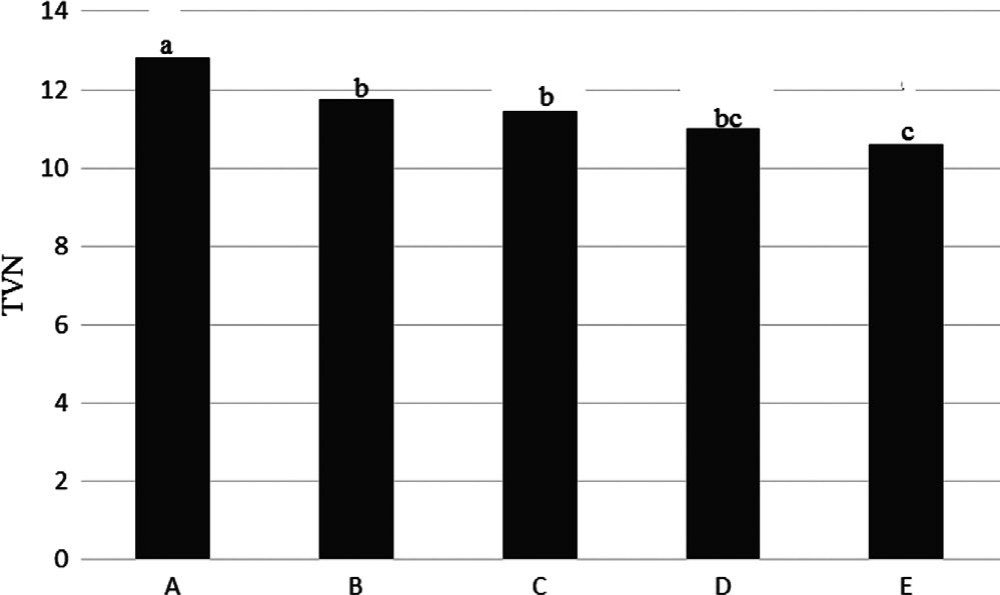
TVN level in the Kotr fish in all treatments during freezing

Table 1 and Figure 9 showed that, total volatile nitrogen (TVN) index had highest value in whole fish (sample A) with 12.81 ± 0.40 mgN/100 g but salted 12% fish (sample E) had lowest value with (10.61 ± 0.38 mgN/100 g). Its amount in some samples had significantly different from each other (*p* < .05).

**Figure 10:**
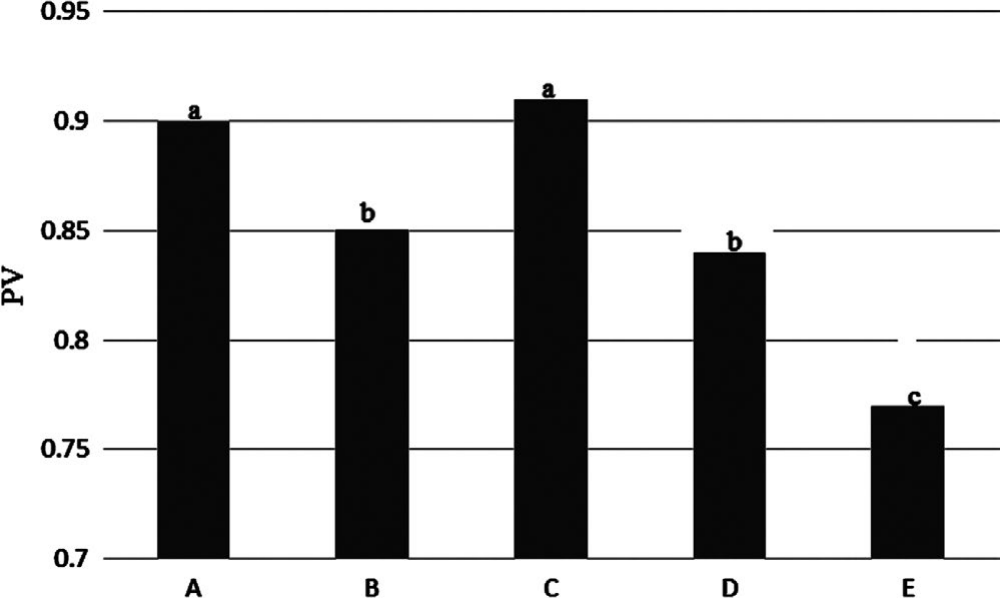
Peroxide values in the Kotr fish in all treatments during freezing

Table 1 and Figure 10, showed peroxide number in the Kotr fish. The highest value of this index was reported in samples A and C, with 0.90 ± 0.02 meq/kg and 0.91 ± 0.02 meq/kg respectively, and sample E had lowest with 0.77 ± 0.02 meq/kg. Amount of this index in some samples had a significant difference with each other (*p* < .05).

**Figure 11:**
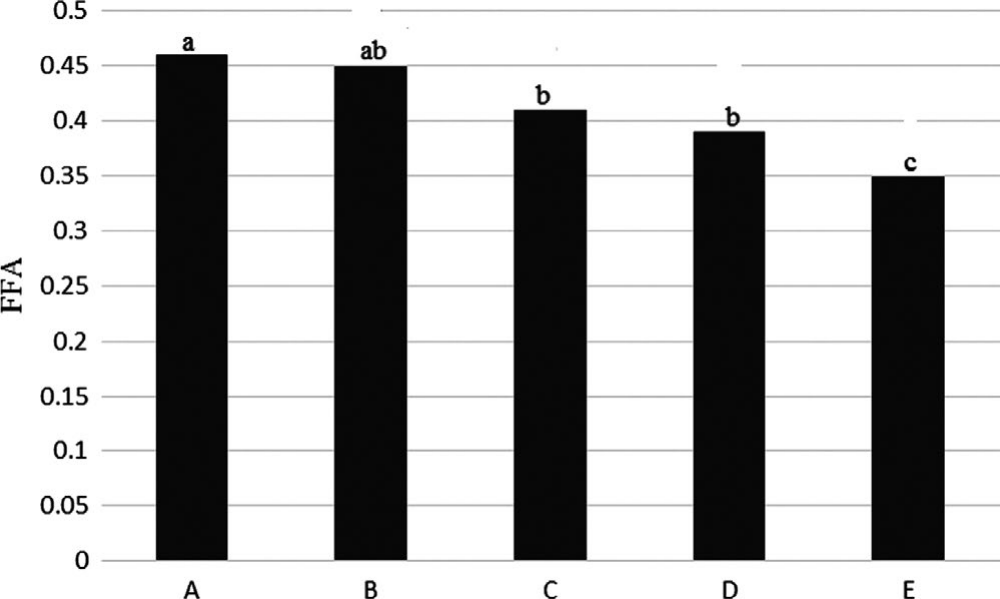
FFA content in the Kotr fish in all treatments during freezing

The results showed that highest of free fatty acids reported for whole fish (sample A) with 0.46 ± 0.04% and lowest amount was for sample E with 0.35 ± 0.01%. Amount of this index in some samples had a significant difference with each other (*p* < .05), which showed in Table 1 and Figure 11.

### 3.8 Proximate composition

There was not a significant difference between crude protein content in the Kotr fish tissue in different samples (*p* < .05). C treatment with (19.81 ± 0.04%) had highest percentage and lowest percentage was for D treatment with (19.66 ± 0.18%) (Table 2).

**Table 2:** Proximate composition of the Kotr fish fillet during freezing in different methods (%)

Proximate composition	A	B	C	D	E
Protein	19.80 ± 0.55^a^	19.68 ± 0.46^a^	19.81 ± 0.04^a^	19.66 ± 0.18^a^	19.71 ± 0.16^a^
Fat	3.64 ± 0.11^a^	3.43 ± 0.19^ab^	3.41 ± 0.06^ab^	3.21 ± 0.05^bc^	3.07 ± 0.22^c^
Moisture	74.90 ± 0.31^a^	75.28 ± 0.32^a^	75.22 ± 0.03^a^	74.90 ± 0.27^a^	75.26 ± 0.04^a^
Ash	2.51 ± 0.15^a^	2.52 ± 0.05^a^	2.38 ± 0.05^a^	2.53 ± 0.34^a^	2.36 ± 0.07^a^
Energetic values (Kcal/100 g)	117.38	114.90	115.35	112.90	111.85

Note

Sample A, was frozen whole fish after washing, sample B, was emptied fish and frozen without saltwater, and samples C, D, and E for emptied fish in saltwater with 4%, 8% and 12% concentration, which frozen respectively. Similar letters in each row indicated no significant difference (*p* < .05).

The during 30 days of freezing period, amount of fat in the Kotr fish different samples had significant difference with each other. Whole fish (sample A) with (3.64 ± 0.11%), had highest percentage and sample E, (3.07 ± 0.22%), had lowest percentage of fat.

There was not a significant different between amount of moisture in the Kotr fish tissue in different treatments (*p* < .05).

Percentage of ash in all samples did not show a significant difference with each other. The different process methods did not effect on amount of ash. However, highest percentage of ash belonged to sample D (2.53 ± 0.34%) and lowest amount belonged to sample E (2.36 ± 0.07%).

The Table 3 showed that number of sycrophile bacteria in whole fish had highest but lowest belonged to sample E (12% salt fish). Depending on process method, sycrophile bacteria load in tissue of the Kotr fish changed significantly (*p* < .05). Treatment A had a significant different comparing to the other treatments (*p* < .05).

**Table 3:** Microbial load of Kotr fish fillets during freezing (CFU/g)

Treatments	A	B	C	D	E
Sycrophile bacteria load	158 × 10^3^ ± 72^a^	115 × 10^3^ ± 44.50^ab^	65 × 10^3^ ± 3^bc^	50 × 10^3^ ± 5^bc^	17 × 10^3^ ± 4.5^c^

Note

Similar letters in each row indicated no significant difference (*p* < .05).

Preservation of fish quality and seafood at its best is most important issue in fish processing. Among various methods of preparation before storage, methods such as emptying the viscera, filleting and grinding of fish is more important in improvement the shelf life of product (Chytiri et al., 2004).

### 3.9 Fatty acid profile

Pirestani et al. (2010) studied on changes in of fatty acids content of several fish species (*R. f. kutum, Liza aurata, Cyprinus carpio, Sander Lucioperca* and *Clupeonella cultiventris caspia*) during storage in freezer. The PUFA decreased a significantly. Also percentage of SFA increased. The Shokri et al. (2015) reported that viscera emptying of the fish rainbow trout resulted to a change in amount of some fatty acids comparing to the frozen whole fish, which was agreement with present study. Shokri et al. (2015) showed that after a 30 days of freezing of the fish rainbow trout, the PUFA/SFA ratio in emptied viscera the fish was higher than that of whole the fish, which was not agreement with present study. DHA and EPA are fatty acids are considered as omega‐3 fatty acids (Feliz et al., 2002). In present study, ratio between DHA/EPA in sample E (salt fish 12%) and sample D (salt fish 8%) was higher than the other treatments.

The 5% NaCl‐treated samples exhibited a significant increase in the total MUFA in *Thunnus tongol* fish (Guizani et al., 2014), which was agreement with present study.

SFA, MUFA, and PUFA in treatment C had highest. PUFA in treatments D and E also were high but there was not a significant different (*p* < .05). ∑ Omega 3 and omega 6 fatty acids in in treatment C was highest, but in treatments of D and E was high that there was not a significant different (*p* < .05). The n3/n6 fatty acids ratio in treatment C was highest. PUFA/SFA ratio in treatment C was high, but more than treatments A and B. Therefore, treatment C was best treatment from point of fatty acids profile comparing to the tother treatments.

### 3.10 Spoilage indexes

The during fish storage, pH increases, which may be due to production of alkaline compounds such as ammonia, trimethylamine, and other biogenic amines produced by spoilage bacteria and endogenous enzymes in fish (Gram & Huss, 1996). A pH level higher than 7.9 can be related as spoilage, and higher than it, leads to lower quality and unusable of fish product (Abroumand et al., 2017). In this study, the pH level in the all samples, except emptied viscera fish, did not show a significant difference. The sample B (emptied viscera fish) had highest level. The Khoda Nazari and Porashuri (2016) and Lokuruka et al. (2012) showed that pH in whole and viscera emptied fish samples during storage in low temperature were not show a significant different which was agreement with present study, but our study showed that pH in the Kotr fish immersed in saltwater was not increased.

Total volatile nitrogen is one of quality indexes of fishery products, bacterial spoilage of fish and enzymatic activities to evaluate quality of these products (Kilinc et al., 2009). This index is widely used as an indexes and degree of lipid oxidation, which can be used to determine level of spoilage and fish useful shelf life. Little increase of this index in initial stages of storage is due to degradation of amino acids and nucleoids, while its increase in final stages of storage is due to increased microbial activities (Sallam et al., 2006). In present study, TVN level in salted samples was lower than in the other samples. Also, its value in abdominal emptied sample was lower than the whole fish. The with addition salt to the Kotr fish fillet, amount of TVN decreased and product shelf life increased. It was clear that amount of TVN in all treatments was lower than allowable limit for human consumption.

Gulyavuz and Ünlüsayýn (1999) stated that a high percentage of salt can be considered as main reason for increase in oxidation process, because salt in high concentrations increases fat oxidation by increase the activity of oxidase enzymes. Maryam et al. (2011) with studies on shelf life of salted mullet fish and packed in vacuum at a temperature of 4°C reported an increase in this index. These results were agreement with Asgharzadeh's work (2001) on effects of salt on shelf life of silver carp. While, in present study, peroxide value in treatments decreased with increase salt concentration. Peroxide index value in whole fish was higher than the other treatments. Fat oxidation is one of major problems in seafood, especially high‐fat foods, which lead to unpleasant taste and smell. An increase in peroxide value to more than 5 milliequivalents per kg of fat, is start of a decrease in quality loss in fish fillets (Karacam & Boran, 1996) reported recommended limit of peroxide value in fish fillets for human consumption was 10 meq per kg of fat (Lodasa et al., 2004). In present study, peroxide number in all samples was lower than recommended level for human consumption.

Thiobarbituric acid index is one of indexes that is used to measure oxidation of fats fillets. The using of sodium salts of organic acids reduces production of TBA in emptied abdominal fish, which shows positive effects of these salts on decrease of oxidation rate during storage at freezer temperature (Kashiri et al., 2011). In present study, level of TBA in the whole fish was higher than the other experiment samples. In salted treatments, amount of this index was less than the other samples and decreased with increase the salt concentration. Porashouri et al. (2015) stated that TVB‐N levels in *R. f. kutum* salted samples were lower than in raw samples, which shown the inhibitory effects of salt on product spoilage. Khoda Nazari and Porashuri (2016) and Viji and et al. (2015) also reported that TBA content in whole fish was higher than emptied abdominal fish, which was agreement with present study. High levels of TBA in whole fish can be due to presence of viscera, which was accumulation place of bacteria that produce enzymes that accelerate oxidative activities.

Atayeter and Ercoşkun (2011) reported that peroxides value (PV) and TBA level increased with higher storage time and temperature for European squid. PV content had an inverse relationship with salt concentration. In the samples treated with 10% salt, peroxide value were significantly higher than samples treated with 5% and 15% salt. Salt at level of 10% accelerates the oxidation of fats and has a protective property in higher concentrations. Rhee and Ziprin (2001) stated that addition of NaCl with 0.5% 2.5% to is a stimulant and thus increase the oxidation of fats. Salt affects the water activity of foods, generally reduces it. The autoxidation of acyl lipids are also affected the activity of water in food. Salt has a strong effect on absorption of water by protein molecules. One of factors determining behavior of fish fillets in different salting media is NaCl interaction with protein matrix (Offer & Trinick, 1983). The state of proteins in the fish muscle has been found to be mainly related to salt concentration of its water phase (Barat et al., 2002). At low salt concentrations, maximum muscle swelling occurs as a result of high water‐protein interaction (highest water holding capacity). At higher salt concentrations, proteins may have strong protein‐protein bonds, resulting in dehydration (Gallart‐Jornet et al., 2007). Some enzymes may be lost during preparation processes; such as emptying abdominal, filleting, and washing, but remained enzymes were in contact with muscle. When fish are exposed to salt, remained enzymes are reactivated and thus lipid hydrolysis occurs, which lead to formation of free fatty acids (Karungi et al., 2004). The results of present study showed that highest amount of FFA belonged to whole fish but in salted samples, its amount decreased. The level of this index in emptied abdominal sample was also higher than the samples immersed in salt. The study of Khoda et al. (2016) showed that amount of TBA, TVN and FFA in whole shorshe fish was more than emptied abdominal fish, which was agreement with present study. The salt concentration had effects on the increase of lipid oxidation (PV and TBA values). The effect was inversely proportional to salt concentration; the highest PV and TBARS values were obtained in the 10% NaCl‐treated samples during the 27 days of storage (Guizani et al., 2014), so that present study results showed TVN, TBA, PV, FFA values were decreased in treatments D and E with 7% and 12% salt concentrations. Energetic value in treatments D and E with 7% and 12% salt concentrations found lower than treatments A, B and C. Protein and fat contents in treatments D and E were decreased.

Moretti et al. (2016) showed that fatty acid composition changed during ripening, with significant increase of saturated fatty acids and decrease of monounsaturated fatty acids. The total concentration of volatile compounds increased rapidly from fresh fish to dried product, with a maximum peak in correspondence to the drying step. The TBA values increased from 0.76 lg/g in fresh fish to 10.95 lg/g in dry fish. Lipolytic and proteolytic reactions occurring in salted product, exacerbated by the presence of high amount of salt and ambient ripening temperature, contributed to the development of typical flavors.

### 3.11 Proximate composition

Although in present study, percentage of protein in treatments had not a significant different, but was agreement with results of study of Khosravi Zadeh and Romiani (2019). They stated that amount of protein in whole shank after one month of storage at −18°C was higher than emptied abdominal Shanak fish, which was agreement with present study. The amount of fat in emptied abdominal the shanak fish was higher than in the whole fish, which was not agreement to present study. Moisture and ash content in the whole shank fish was higher than emptied abdominal the fish, which not agreement with present study. Results of Shokri et al. (2015) showed that abdominal emptying did not lead to a significant change in amount of ash and protein between two the treatments.

### 3.12 Sycrophile bacteria

Gram negative sycrophile bacteria are main group of microorganisms responsible for spoilage of freshly stored fish at refrigerated temperatures (Huss, 1996). Results showed that number of sycrophile bacterial colonies in whole catfish after 30 days storage in freezing at −18°C was higher than the other samples, while salted 12% sample had lowest microbial load. The emptying abdominal, washing and increase salt concentration showed a significant decrease in bacterial load of sycrophile, which indicated slow growth of sycrophile bacteria and thus, increase shelf life of the Kotr fish at −18°C. In present study, there was no a significant difference between number of sycrophile bacteria in the whole fish and the emptied abdominal fish, which was agreement with results of Shokri et al. (2015). They stated that abdominal emptying of the fish rainbow trout did not show a significant change in population of sycrophile bacteria.

In present study, effects of abdominal emptying and immersion in salt solution with different concentrations on fatty acid profile and spoilage indexes of *S. jello* fillet during freezing at −18°C was investigated. It can be concluded that abdominal emptying, washing, and immersion of the Kotr fish in saltwater lead to an increase in percentage of SFA in the fish fillet during freezing, although, amount of MUFA, PUFA, omega 3 and omega 6 fatty acids did not show a significant different between different treatments, but percentage of MUFA in salted 4% fish and percentage of PUFA and omega 3 fatty acids as well as ratio of omega 3 to omega 6 fatty acids in salted all samples was higher than the other samples. The spoilage indexes in the Kotr fish fillet during freezing under influence of different process methods had a significant difference with each other and amount of all indexes significantly reduced with fish immersion in saltwater. The abdominal emptying and immersion of the Kotr fish in saltwater except fat did not show a significant effect on other proximate composition of fish fillet but there was not a significant difference. The abdominal emptying and addition of salt to fish fillet lead to a significant decrease in microbial load of the Kotr fish during freezing. It can be concluded that immersion fish in saltwater lead to improvement the indexes of spoilage, decrease the bacterial load and preservation the nutritional value of the Kotr fish during the 30 days of freezing at −18°C.

## 4 CONCLUSIONS

The results of present study showed that percentage of SFA in whole fish was significantly lower than the other treatments, but percentage of omega 3, omega 6 fatty acids and MUFA and PUFA in different samples did not show a significant difference, but ratio between DHA/EPA fatty acids significantly changed. The value of spoilage indices in *S. jello* fillet were different in process different methods but amount of spoilage indices reduced with addition salt. Addition salt and emptying abdominal of the *S. jello* lead to a decrease in the fat content of the fish fillet, but there was not a significant effect on protein, moisture and ash content. The number of cyclophil bacteria was 158 × 10^3^ Cfu/g in treatment of A but it deceased in the other treatments. It can be concluded that abdominal emptying and immersion of the Kotr fish in saltwater before freezing can lead to preservation of the fish nutritional value and increase in shelf life of product.

## CONFLICTS OF INTEREST

The authors do not have any conflict of interest.

## ETHICAL APPROVAL

This study did not involve human or animal testing.

## INFORMED CONSENT

Written informed consent was obtained from all study participants.
